# Protecting aircrew from cold stress elevates heat stress

**DOI:** 10.1186/2046-7648-4-S1-A18

**Published:** 2015-09-14

**Authors:** Andrew P Hunt

**Affiliations:** 1 Land Division, Defence Science and Technology Organisation, Melbourne, Australia

## Introduction

Aircrew need to wear an immersion suit to protect against hypothermia in the unlikely event of crashing into cold water. These clothing configurations provide insulation and water tight seals to reduce body heat loss in water; however, heat loss will also be impaired during normal flying activities in a warm cockpit. Therefore, this study evaluated the heat exchange properties of the Aircrew Protective Clothing Configuration (APCC) to determine the limitations it may impose to work in warm environments.

## Methods

The heat exchange properties of six APCC's were measured on a heated sweating manikin (Newton P-352, MTNW, USA) in accordance with standard test procedures [1,2]. Outer garments included a standard flying suit (FLY) and a Constant Wear Immersion Suit (CWIS). Three combinations of undergarments were evaluated with each outer garment. The Method for Evaluating Thermal Strain (METHS) model was used to estimate the heat strain of wearing the APCC's. The work scenario included a 20 min pre-flight period of moderate work (350 W) and a flight period of up to 4 hours of light work (150 W). The work duration until core body temperature rose from 37.0 °C to 38.5 °C was examined across a range of Wet-Bulb Globe Temperature (WBGT) including: 23.6, 26.4, 26.9, 28.5, 29.5, 29.7, 31.1, 32.5, 33.0, and 36.5 °C.

## Results

**Figure 1:**
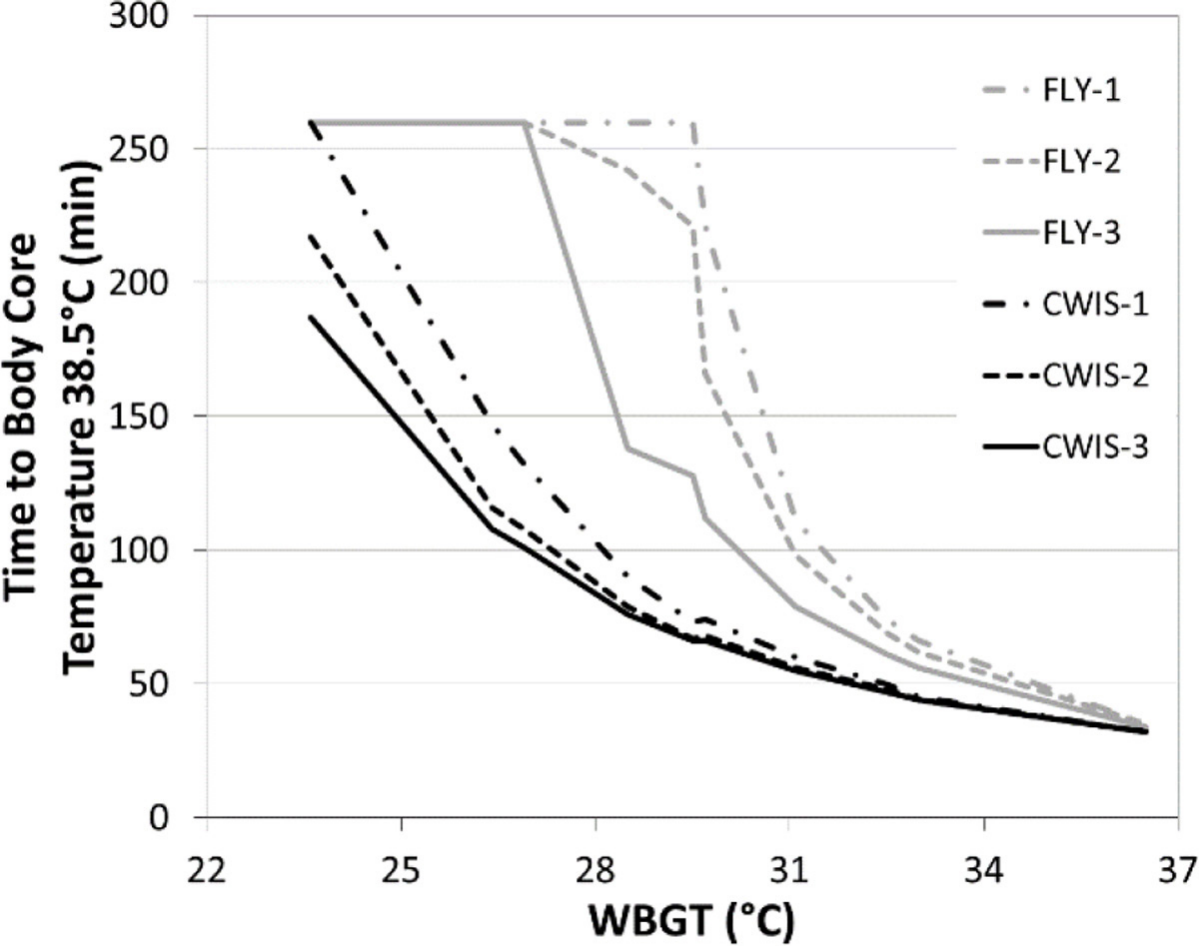
The time to body core temperature of 38.5 °C across a range in WBGT for each clothing configuration.

Additional undergarments increased the thermal resistance of the FLY and CWIS configurations (FLY-1: 0.234; FLY-2: 0.269, and FLY-3: 0.320; CWIS-1: 0.273; CWIS-2: 0.317; and CWIS-3: 0.334 °C.m^2^.W^-1^). The CWIS configurations had a distinctly higher evaporative resistance than the FLY configurations, which also increased with additional undergarments (FLY-1: 0.039; FLY-2: 0.043; FLY-3: 0.051; CWIS-1: 0.062; CWIS-2: 0.070; CWIS-3: 0.073 kPa.m^2^.W^-1^). Work duration in the WBGT range of 24-30 °C was much shorter for the CWIS compared to the FLY ensembles (Figure 1). Alternatively, in the most oppressive conditions (WBGT 32.0-36.5 °C) the work duration was similar in either the FLY or CWIS configurations (Figure 1).

## Discussion

Protecting aircrew from hypothermia will elevate heat strain and restrict work duration in the WBGT range of 24-30 °C compared to wearing standard flight suits. However, in warmer conditions the choice of APCC had a negligible effect on work duration, which was highly restricted irrespective of the clothing worn.

## Conclusion

The decision to don immersion protective clothing to protect against hypothermia in the event of a crash into cold water must be balanced with the risk of elevated heat strain during flight.

## References

[B1] American Society for Testing and Materials Standard Test Method for Measuring Thermal Insulation of Clothing Using a Heated Manikin ASTM 2010 F1291 10

[B2] American Society for Testing and Materials Standard Test Method for Measuring the Evaporative Resistance of Clothing Using a Sweating Manikin ASTM 2010 F2370 10

